# Well-Being Parameters and Intention to Leave Current Institution Among Academic Physicians

**DOI:** 10.1001/jamanetworkopen.2023.47894

**Published:** 2023-12-15

**Authors:** Jennifer A. Ligibel, Nicolette Goularte, Jennifer I. Berliner, Steven B. Bird, Chantal M. L. R. Brazeau, Susannah G. Rowe, Miriam T. Stewart, Mickey T. Trockel

**Affiliations:** 1 Department of Medical Oncology, Dana-Farber Cancer Institute, Boston, Massachusetts; 2 WellMD & WellPhD, Stanford University School of Medicine, Stanford, California; 3 Division of Cardiology and The Heart and Vascular Institute, Department of Medicine, University of Pittsburgh School of Medicine, Pittsburgh, Pennsylvania; 4 Department of Emergency Medicine, UMass Chan Medical School, Worcester, Massachusetts; 5 Rutgers Biomedical and Health Sciences, Newark, New Jersey; 6 Department of Ophthalmology, Boston University Chobanian & Avedisian School of Medicine, Boston, Massachusetts; 7 Office of Equity, Vitality and Inclusion, Boston Medical Center, Boston University Medical Group, Boston, Massachusetts; 8 Division of General Pediatrics, Perelman School of Medicine of the University of Pennsylvania, Philadelphia

## Abstract

**Importance:**

Physician turnover interrupts care delivery and creates health care system financial burden.

**Objective:**

To describe the prevalence of burnout, professional fulfillment, and intention to leave (ITL) among physicians at academic-affiliated health care systems and identify institutional and individual factors associated with ITL.

**Design, Setting, and Participants:**

This cross-sectional study administered a survey to 37 511 attending-level medical specialists at 15 academic medical institutions participating in the Healthcare Professional Well-Being Academic Consortium. Data were collected from October 2019 to July 2021. Statistical analysis was performed from May 2022 to March 2023.

**Exposures:**

Hypothesized institutional and individual determinants of occupational well-being.

**Main Outcomes and Measures:**

The main outcome was ITL, defined as having at least a moderate intention (a score of 2 on a 0-4 scale) to leave one’s institution within the next 2 years. Additional outcomes included burnout and professional fulfillment, defined using published Professional Fulfillment Index cut points.

**Results:**

Of 18 719 academic physician survey respondents (8381 [44.8%] male; 2388 [12.8%] Asian, 10 599 [56.6%] White, 1039 [5.6%] other race, 4693 [25.1%] unknown race; 294 [1.6%] Hispanic or Latina/Latino/Latinx), 6903 of 18 217 (37.9%) met criteria for burnout and 7301 of 18 571 (39.3%) for professional fulfillment; 5177 of 15 890 (32.6%) reported moderate or greater ITL. Burnout, professional fulfillment, and ITL varied across specialties. After adjusting for demographics, each 1-point increase (range 0-10) in burnout was directly associated with ITL (odds ratio [OR], 1.52 [95% CI, 1.49-1.55])c, and each 1-point increase in professional fulfillment was inversely associated with ITL (OR, 0.64 [95% CI, 0.63-0.65]). After adjusting for demographics, burnout, and professional fulfillment, each 1-point increase (range 0-10) in supportive leadership behaviors (OR, 0.83 [95% CI, 0.82-0.84]), peer support (OR, 0.93 [95% CI, 0.91-0.95]), personal-organizational values alignment (OR, 0.81 [95% CI, 0.80-0.82]), perceived gratitude (OR, 0.95 [95% CI, 0.92-0.97]), COVID-19 organizational support (OR, 0.88 [95% CI, 0.85-0.91]), and electronic health record helpfulness (OR, 0.95 [95% CI, 0.93-0.97]) were inversely associated with ITL, whereas each 1-point increase (range 0-10) in depression (OR, 1.08 [95% CI, 1.05-1.10]) and negative impact of work on personal relationships (OR, 1.09 [1.07-1.11]) were directly associated with ITL.

**Conclusions and Relevance:**

In this cross-sectional study of academic physicians, 32.6% indicated moderate or higher ITL within 2 years. Burnout, lack of professional fulfillment, and other well-being factors were associated with ITL, suggesting the need for a comprehensive approach to reduce physician turnover.

## Key Points

**Question:**

What proportion of academic physicians intend to leave their current institution within the next 2 years, and what factors are associated with intention to leave?

**Findings:**

In this cross-sectional study of 18 719 academic physicians, approximately one-third reported moderate or greater intention to leave. Burnout, lack of professional fulfillment, and other personal and organizational factors were associated with intention to leave.

**Meaning:**

In this study, intention to leave was prevalent in academic physicians and was associated with numerous well-being factors; a comprehensive approach will be needed to reduce physician turnover.

## Teaser

This cross-sectional study describes the prevalence of burnout, professional fulfillment, and intention to leave among physicians at academic-affiliated health care systems and identifies institutional and individual factors associated with intention to leave.

## Introduction

Physician departures from clinical settings interrupt therapeutic and professional relationships, disrupt care delivery, and shift the burden of clinical care to remaining members of the health care team. In 2020, the Association of American Medical Colleges (AAMC) reported that 6% to 7% of the physician workforce left practice settings each year. Recent data suggest that physician turnover has increased substantially; a 2022 survey of more than 500 physicians by CHG Healthcare found that 43% of survey respondents had changed jobs over the course of the prior 2 years, including 8% who retired and 3% who left medicine to pursue nonclinical careers.^1^ Another 2023 survey of 500 physicians by the Massachusetts Medical Society found that 27% of survey respondents indicated that they would “definitely” or “likely” leave medicine within the next 2 years, suggesting that high rates of physician turnover are likely to continue.

Physicians who leave medicine not only disrupt care at their institutions, but also contribute to physician shortages. The AAMC estimates that the US will face a shortage of between 37 800 and 124 000 physicians by 2034.^2^ Projected shortages differ by specialty, with an expected gap of 17 800 to 48 000 primary care physicians, 15 800 to 30 200 surgical specialists, and 3800 to 13 400 medical specialists, suggesting that attrition of some segments of the physician population could further exacerbate these anticipated shortages and have a disproportionate effect on care delivery.

Prior studies have shown that burnout is associated with higher physician turnover. A cross-sectional study of 1840 health professionals (including physicians, nurses, and midwives) employed by public hospitals and rehabilitation clinics in Switzerland found a significant association between burnout and thoughts of leaving medicine.^3^ Work-life imbalance was the strongest predictor of burnout symptoms among physicians, but effort-reward mismatch was the strongest predictor for having thoughts of leaving the medical profession. Another study looking at burnout and physician attrition in a cohort of 472 physicians at 2 Stanford University system hospitals who had completed a physician wellness survey reported that individuals who met criteria for burnout were more than twice as likely to have left the institution over the ensuing 2 years as compared with individuals who did not meet criteria for burnout^4^; importantly, in this study, individuals who reported moderate or greater intention to leave (ITL) the institution within the next 2 years on the baseline survey were at significantly increased risk of attrition during the ensuing 2 years compared with those who did not endorse ITL (relative risk [RR], 3.2 [95% CI, 2.0-5.1]),^4^ suggesting that ITL is a significant risk factor for subsequent physician turnover.

Although burnout has been shown to be associated with both ITL and actual turnover, less is known about other contributing factors. Additionally, there is limited information regarding differences in ITL among medical specialties. The Healthcare Professional Well-being Academic Consortium (PWAC) is a self-funded group of academic affiliated health care organizations using common metrics to assess professional fulfillment, burnout, and their hypothesized determinants in clinicians. In this study, we describe the prevalence of burnout, professional fulfillment, and ITL by specialty across 15 academic sites that administered the PWAC survey between 2019 and 2021, explore differences in ITL between medical specialties, and identify individual and institutional factors associated with ITL.

## Methods

Data for this cross-sectional study were collected by 15 organizations participating in the PWAC that administered a survey to assess professional fulfillment, burnout, and their hypothesized determinants in physicians between October 2019 and July 2021 (eTables 1 and 2 in Supplement 1). All practicing physicians in participating organizations were invited to complete the survey. Surveys were voluntary and participants were permitted to skip questions they preferred not to answer or that did not apply. Surveys were streamlined to include the same core measures (burnout, professional fulfillment, ITL, and demographics) and also customized to allow individual sites to assess hypothesized proximal determinants of well-being that aligned with their unique program goals and action areas. Surveys were distributed via individual email to each participant with up to 6 reminders for individuals who had not yet completed the Professional Fulfillment Index (PFI), the survey’s primary outcome measure.

An independent professional survey vendor conducted all surveys. The survey vendor performed deidentification to remove potentially identifying information, including combinations of demographics and medical specialty information unique to any group of fewer than 5 respondents. Organizational identifiers were also removed from the aggregate data so respondents could not be connected to their employer. For this cross-sectional study, the deidentification process prioritized retention of demographic and practice specialty information in the following order: medical practice specialty, gender, race, ethnicity, and age category. Subspecialties with fewer than 100 respondents were included in the report of the parent primary specialty, along with responses indicating no subspecialty. The aggregate data set includes all core measures, and all other survey measures used by at least 4 institutions. The Stanford institutional review board deemed this study exempt as it uses deidentified, administratively collected data. We followed the Strengthening the Reporting of Observational Studies in Epidemiology (STROBE) reporting guideline for cross-sectional studies to develop the analytic plan and reporting structure for this study.

### Measures

PWAC selection of measures to assess occupational well-being was guided by a proposed model of reciprocal causation between cultural factors, work efficiency factors, and individual physician factors, all of which are hypothesized to contribute to clinician occupational well-being.^5^ This proposed model is consistent with reciprocal determinism principle of social cognitive theory.^6^ Burnout and professional fulfillment were assessed using the PFI, which has demonstrated reliability,^7,8^ validity,^7,8^ and sensitivity to change.^8^ Published cut points were used to dichotomize burnout (score ≥3.325; range: 0 to 10 [higher scores indicate more burnout]) and professional fulfillment (score ≥7.5; range: 0 to 10 [higher scores indicate more professional fulfillment]) to estimate point-prevalence of both (see eMethods in Supplement 1). Intention to leave was assessed with a standard question that asks respondents to indicate the likelihood of leaving their current institution within 2 years, with 5 Likert scale response options from “none” to “definitely.” Consistent with a previous report, responses of “moderate,” “likely,” and “definitely” were classified as “intent to leave.”^4^

All assessments of hypothesized determinants of professional fulfillment and burnout used 5-point Likert scale items (eTable 3 in Supplement 1). Measures of personal-organizational values alignment,^9^ control over schedule, negative impact of work on personal relationships,^10^ meaningfulness of clinical work, and peer support were developed at Stanford University concurrent with development of the PFI, with iterative review by a group of experts in physician well-being to establish face and content validity.^8^ Measures of self-valuation^11,12^ (ie, self-compassion), perceived gratitude,^13^ electronic health records (EHR) helpfulness, and EHR hassles were developed subsequently using similar methods. The COVID-19 organizational support scale was developed to assess key areas of support that focus groups of physicians indicated were important.^14^ Supportive leadership behaviors were assessed using the Participatory Management Leadership Index.^9,15^ The Patient-Reported Outcomes Measurement Information System short-form 4-item versions were used to assess depression, anxiety, and sleep-related impairment.^16,17,18^ All scale scores used as independent variables in analyses were normalized to a 0 to 10-point range to facilitate interpretation.

### Statistical Analysis

Analyses were performed from May 2022 to March 2023 using SPSS version 28.0.1.1 (IBM Corp). A response was defined as answering at least 75% of items within 1 or more outcome measures (burnout, professional fulfillment, and ITL). An individual respondent’s data were included in the analyses if at least 75% of items within each survey measure were answered.

Standard descriptive summary statistics including mean (SD) were used to describe sample characteristics. χ^2^ Tests of independence were used to evaluate differences in ITL across gender and race. We specified a multivariable logistic regression model adjusting for self-reported gender (reference group: male), self-reported subspecialty (reference group: internal medicine), self-reported age (reference group: 40-59 years), self-reported ethnicity (reference group: not Hispanic), and self-reported race (reference group: White) to assess the association of ITL with burnout and professional fulfillment. We elected to adjust for these demographic variables because of their known association with burnout.

An additional series of logistic regression models were used to assess the associations between ITL and proximal determinants of well-being: (1) sleep-related impairment, (2) supportive leadership behaviors, (3) control over schedule, (4) peer support, (5) negative impact of work on personal relationships, (6) self-valuation, (7) meaningfulness of clinical work, (8) personal-organizational values alignment, (9) perceived gratitude, (10) COVID-19 organizational support, (11) anxiety, (12) depression, (13) EHR helpfulness, and (14) EHR hassles. While each model 0 is unadjusted, each model 1 is adjusted for gender, subspecialty, age, ethnicity, and race. Each model 2 is adjusted for all demographics in model 1, as well as professional fulfillment and burnout. Loss of data due to missingness was minimized by including the category of missing in analyses when adding categorical variables to regression models. Statistical significance for all analyses was 2-sided and set at *P* < .001.

## Results

A total of 18 719 physicians from 15 academic medical institutions completed the PWAC survey between October 2019 and July 2021 and met criteria for inclusion in this analysis. The survey was distributed to 37 511 attending-level medical specialists (eFigure in Supplement 1), and 19 029 individuals met the response criteria by responding to at least 1 outcome measure (>75% of items answered for professional fulfillment, burnout, or ITL) resulting in an overall survey response rate of 50.7%. Nonphysician medical specialists (dentistry, clinical psychology, podiatry, and medical physics) were removed, resulting in an analytic cohort of 18 719 physicians. Overall, 8381 (44.8%) were male, 8037 (42.9%) were female, 2301 (12.3%) did not report their gender or reported a gender identification whose prevalence was below the deidentification threshold; 2388 (12.8%) were Asian, 10 599 (56.6%) were White, 1039 (5.6%) were other races, and 4693 (25.1%) were missing race data; 294 (1.6%) were Hispanic or Latina/Latino/Latinx, 13 114 (70.1%) were not Hispanic or Latina/Latino/Latinx, and 5311 (28.4%) were missing ethnicity data (Table 1). Survey respondents represented 53 medical specialties.

**Table 1.** Characteristics of Survey Respondents

Characteristics	Respondents, No. (%) (N = 18 719)
Gender	
Female	8037 (42.9)
Male	8381 (44.8)
Missing or gender identification other than male or female	2301 (12.3)
Age, y	
≤39	3915 (20.9)
40-59	8855 (47.3)
≥60	2532 (13.5)
Missing	3417 (18.3)
Ethnicity	
Hispanic or Latina/Latino/Latinx	294 (1.6)
Not Hispanic or Latina/Latino/Latinx	13 114 (70.1)
Missing	5311 (28.4)
Race	
Asian	2388 (12.8)
White	10 599 (56.6)
Other^a^	1039 (5.6)
Missing	4693 (25.1)

^a^ Other race category included African American or Black, American Indian or Alaska Native, Native Hawaiian or Pacific Islander, prefer to self-describe (write-ins), and combinations of more than 1 race option.

### ITL in Study Cohort as a Whole and by Subspeciality

Of 18 719 survey respondents, 15 890 (84.9%) elected to respond to the ITL item. Of these, 5177 (32.6%) reported moderate or greater ITL (Table 2). ITL varied modestly by gender, with 2204 of 7073 women (31.2%) and 2493 of 7548 men (33.0%) reporting ITL (*P* = .02). ITL also varied by race, with 558 of 1932 Asian individuals (28.9%), 3000 of 9606 White individuals (31.2%), and 357 of 918 individuals with other race (38.9%) reporting ITL (*P* < .001); and by age, with 921 of 2297 individuals (40.1%) aged 60 years and older, 2321 of 7928 (29.3%) aged 40 to 59 years, and 1127 of 3469 (32.5%) aged 39 years and younger reporting ITL (*P* < .001).

**Table 2.** Prevalence of Burnout, Professional Fulfillment, and Intention to Leave Among Survey Respondents

Characteristics	Professional fulfillment			Burnout			Intention to leave		
	Total No. with data	Professional fulfillment present, No. (%)	*P* value	Total No. with data	Burnout present, No. (%)	*P* value	Total No. with data	Moderate or higher intention to leave, No. (%)	*P* value
Total	18 571	7301 (39.3)	NA	18 217	6903 (37.9)	NA	15 890	5177 (32.6)	NA
Gender									
Female	7979	2724 (34.1)	<.001^a^	7900	3332 (42.2)	<.001^a^	7073	2204 (31.2)	.02^a^
Male	8317	3775 (45.4)		8264	2730 (33.0)		7548	2493 (33.0)	
Missing	2275	802 (35.3)	NA	2053	841 (41.0)	NA	1269	480 (37.8)	NA
Age, y									
≤39	3893	1414 (36.3)	<.001^a^	3882	1577 (40.6)	<.001^a^	3469	1127 (32.5)	<.001^a^
40 to 59	8796	3380 (38.4)		8717	3530 (40.5)		7928	2321 (29.3)	
≥60	2499	1237 (49.5)		2469	572 (23.2)		2297	921 (40.1)	
Missing	3383	1270 (37.5)	NA	3149	1224 (38.9)	NA	2196	808 (36.8)	NA
Ethnicity									
Hispanic or Latina/Latino/Latinx	292	123 (42.1)	.41^a^	292	110 (37.7)	.90^a^	261	81 (31.0)	.91^a^
Not Hispanic or Latina/Latino/Latinx	13 023	5176 (39.7)		12 948	4832 (37.3)		11 710	3674 (31.4)	
Missing	5256	2002 (38.1)	NA	4977	1961 (39.4)	NA	3919	1422 (36.3)	NA
Race									
Asian	2374	996 (42.0)	.003^a^	2359	773 (32.8)	<.001^a^	1932	558 (28.9)	<.001^a^
White	10 520	4237 (40.3)		10 465	3899 (37.3)		9606	3000 (31.2)	
Other^b^	1034	369 (35.7)		1029	413 (40.1)		918	357 (38.9)	
Missing	4643	1699 (36.6)	NA	4364	1818 (41.7)	NA	3434	1262 (36.8)	NA

Abbreviation: NA, not applicable.

^a^ Missing categories were excluded from χ^2^ tests of equivalence across groups.

^b^ Other race category included African American or Black, American Indian or Alaska Native, Native Hawaiian or Pacific Islander, prefer to self-describe (write-ins), and combinations of more than 1 race option.

**Figure 1.**
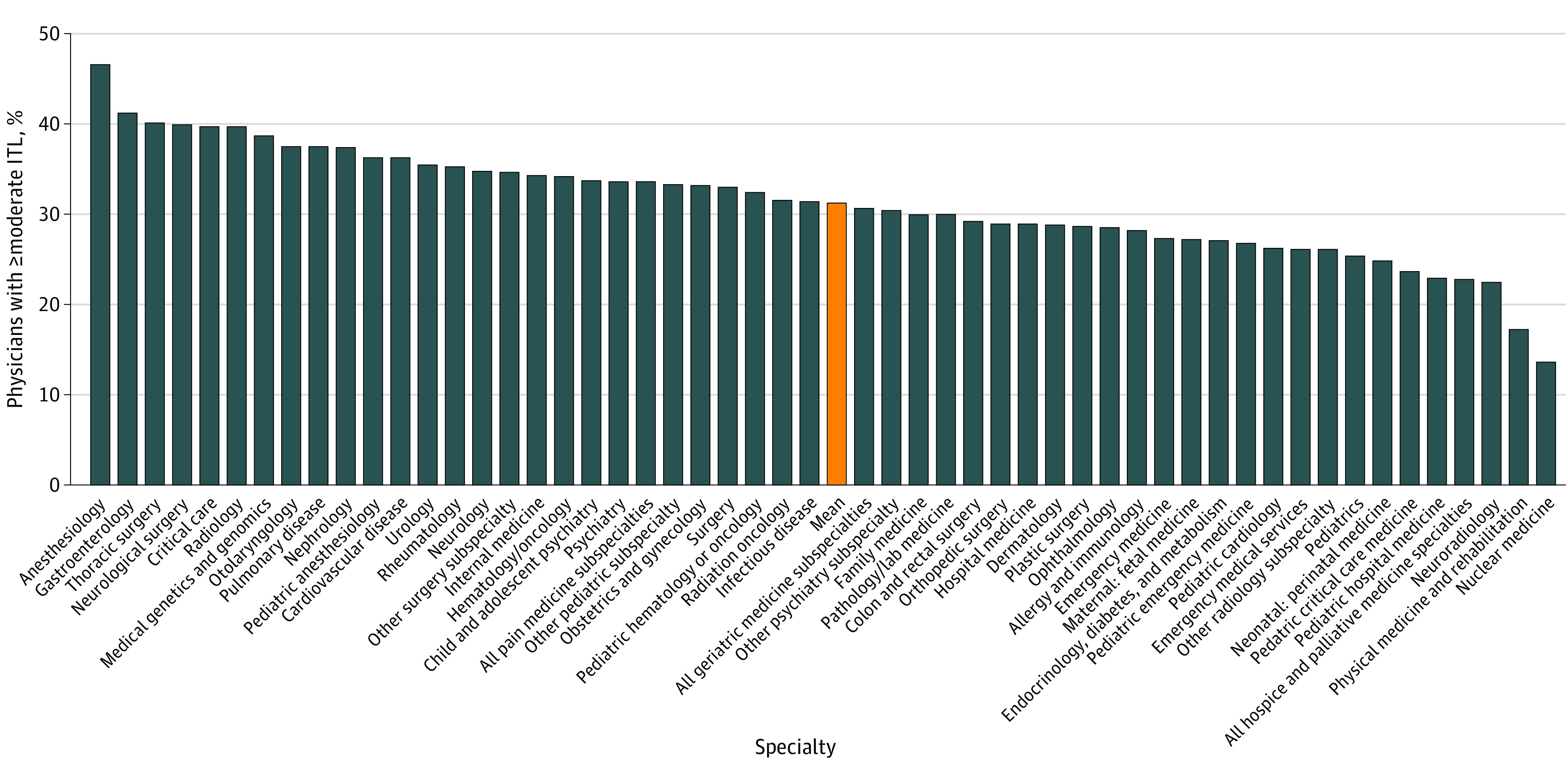
Intention to Leave (ITL) by Medical Specialty

ITL also varied among medical specialties (Figure 1; eTable 4 in Supplement 1). Anesthesiology reported the highest rate of ITL at 46.8% (95% CI, 42.5%-51.0%). Other specialties with high rates of ITL included gastroenterology (41.3% [95% CI, 34.7%-47.9%]), thoracic surgery (40.2% [95% CI, 29.7%-50.7%]), neurological surgery (40.0% [95% CI, 30.0%-50.0%]), critical care (39.8% [95% CI, 33.8%-45.9%]), and radiology (39.8% [95% CI, 33.9%-45.6%]). Nuclear medicine reported the lowest rate of ITL at 13.6% (95% CI, 0.0%-29.2%). Other groups with low rates of ITL included physical medicine and rehabilitation (17.3% [95% CI, 10.7%-24.0%]), neuroradiology (22.6% [95% CI, 15.1%-30.0%]), hospice and palliative care (22.9% [95% CI, 15.8%-29.9%]), and pediatric hospital medicine (23.1% [95% CI, 17.1%-29.0%]).

### Burnout and Professional Fulfillment

In the entire cohort, 6903 of 18 217 (37.9%) met criteria for burnout. The prevalence of burnout differed by gender, with 3332 of 7900 women (42.2%) and 2730 of 8264 men (33.0%) meeting criteria for burnout (*P* < .001). The prevalence of burnout also varied by age with 572 of 2469 survey respondents aged 60 years or older (23.2%), 3530 of 8717 respondents aged 40 to 59 years (40.5%), and 1577 of 3882 respondents aged 39 years or younger (40.6%) meeting criteria for burnout (*P* < .001). Finally, burnout varied by race, with 3899 of 10 465 White (37.3%), 773 of 2359 Asian (32.8%), and 413 of 1029 survey respondents of other races (40.1%) meeting criteria for burnout (*P* < .001).

In total, 7301 of 18 571 respondents (39.3%) met criteria for professional fulfillment. Rates of professional fulfillment varied by gender, with 2724 of 7979 women (34.1%) and 3775 of 8317 men (45.4%) meeting criteria for professional fulfillment (*P* < .001); and by age, with 1237 of 2499 respondents aged 60 years or older (49.5%), 3380 of 8796 respondents aged 40 to 59 years (38.4%), and 1414 of 3893 respondents aged 39 years or younger (36.3%) meeting criteria for professional fulfillment (*P* < .001). Professional fulfillment also varied by race, with 4237 of 10 520 White (40.3%), 996 of 2374 Asian (42.0%), and 369 of 1034 survey respondents of other races (35.7%) meeting criteria for professional fulfillment (*P* = .003).

**Figure 2.**
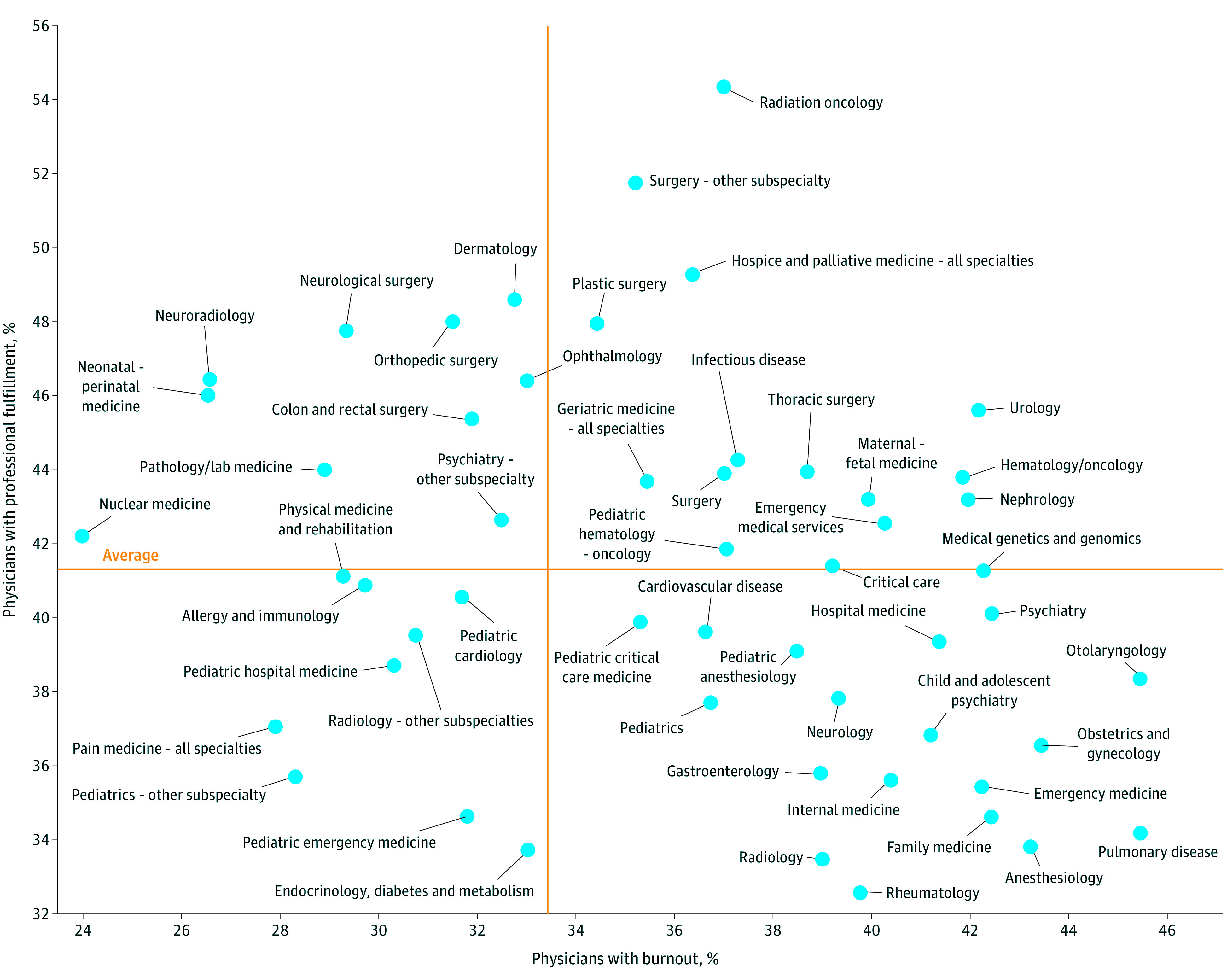
Burnout and Professional Fulfillment by Medical Specialty

The prevalence of burnout and professional fulfillment varied by specialty (Figure 2; eTable 5 in Supplement 1). In some specialties (such as anesthesiology, pulmonary disease, family medicine, and emergency medicine), a relatively high proportion of survey respondents met criteria for burnout, while a relatively low proportion met criteria for professional fulfillment. In other specialties (such as neuroradiology, neurological surgery, and neonatal-perinatal medicine), the proportion who met criteria for professional fulfillment was relatively high and the proportion meeting criteria for burnout was relatively low. Other specialties (such as pain medicine and pediatric subspecialties), reported relatively low levels of burnout and low levels of professional fulfillment, while others (such as urology and hematology/oncology) reported relatively high levels of burnout and professional fulfillment.

### Factors Associated With ITL

In a multivariable model, adjusting for demographic characteristics and medical specialty, each 1-point higher burnout score (range 0-10) was associated with 52% higher odds of ITL (OR, 1.52 [95% CI, 1.49-1.55]). Each 1-point higher professional fulfillment score (range 0-10) was associated with 36% lower odds of ITL (OR, 0.64 [95% CI, 0.63-0.65]).

After adjusting for demographic characteristics, burnout, and professional fulfillment (Table 3), each 1-point higher score (range 0-10) on measures of supportive leadership behaviors (OR, 0.83 [95% CI, 0.82-0.84]), peer support (OR, 0.93 [95% CI, 0.91-0.95]), personal-organizational values alignment (OR, 0.81 [95% CI, 0.80-0.82]), perceived gratitude (OR, 0.95 [95% CI, 0.92-0.97]), COVID-19 organizational support (OR, 0.88 [95% CI, 0.85-0.91]), and EHR helpfulness (OR, 0.95 [95% CI, 0.93-0.97]) were associated with lower ITL, whereas each 1-point higher score (range 0-10) on measures of depression (OR, 1.08 [95% CI, 1.05-1.10]) and negative impact of work on personal relationships (OR, 1.09 [95% CI, 1.07-1.11]) were associated with higher ITL.

**Table 3.** Associations Between Individual and Organizational Factors and Intention to Leave

Independent variable^a^ and model^b^	Odds ratio (95% CI)	*P* value	Bonferroni-adjusted *P* value	Nagelkerke pseudo *R^2^*
Burnout^c^				
Model 0	1.48 (1.45-1.50)	<.001	<.001	0.15
Model 1	1.52 (1.49-1.55)	<.001	<.001	0.19
Model 2a	1.24 (1.20-1.27)	<.001	<.001	0.25
Professional fulfillment^c^				
Model 0	0.66 (0.65-0.67)	<.001	<.001	0.19
Model 1	0.64 (0.63-0.65)	<.001	<.001	0.23
Model 2b	0.72 (0.71-0.74)	<.001	<.001	0.25
Sleep-related impairment (4-item)				
Model 0	1.18 (1.15-1.20)	<.001	<.001	0.04
Model 1	1.19 (1.17-1.21)	<.001	<.001	0.08
Model 2	1.02 (1.00-1.05)	.04	NA	0.25
Supportive leadership behaviors^c^				
Model 0	0.74 (0.73-0.75)	<.001	<.001	0.14
Model 1	0.74 (0.72-0.75)	<.001	<.001	0.17
Model 2	0.83 (0.82-0.84)	<.001	<.001	0.28
Control over schedule				
Model 0	0.85 (0.83-0.86)	<.001	<.001	0.04
Model 1	0.83 (0.81-0.84)	<.001	<.001	0.08
Model 2	0.98 (0.96-1.00)	.05	NA	0.25
Peer support^c^				
Model 0	0.80 (0.79-0.82)	<.001	<.001	0.08
Model 1	0.81 (0.79-0.82)	<.001	<.001	0.12
Model 2	0.93 (0.91-0.95)	<.001	<.001	0.26
Negative impact of work on personal relationships^c^				
Model 0	1.22 (1.20-1.23)	<.001	<.001	0.10
Model 1	1.23 (1.22-1.25)	<.001	<.001	0.14
Model 2	1.09 (1.07-1.11)	<.001	<.001	0.26
Self-valuation				
Model 0	0.82 (0.81-0.84)	<.001	<.001	.05
Model 1	0.81 (0.80-0.83)	<.001	<.001	0.09
Model 2	0.98 (0.96-1.01)	.18	NA	0.26
Meaningfulness of clinical work				
Model 0	0.83 (0.81-0.85)	<.001	<.001	0.04
Model 1	0.82 (0.80-0.84)	<.001	<.001	0.09
Model 2	1.06 (1.03-1.10)	<.001	.003	0.27
Personal-organizational values alignment^c^				
Model 0	0.71 (0.70-0.72)	<.001	<.001	0.20
Model 1	0.71 (0.70-0.72)	<.001	<.001	0.22
Model 2	0.81 (0.80-0.82)	<.001	<.001	0.29
Perceived gratitude^c^				
Model 0	0.79 (0.77-0.80)	<.001	<.001	0.08
Model 1	0.78 (0.77-0.80)	<.001	<.001	0.11
Model 2	0.95 (0.92-0.97)	<.001	<.001	0.26
COVID-19 organizational support^c^				
Model 0	0.77 (0.75-0.80)	<.001	<.001	0.10
Model 1	0.76 (0.74-0.79)	<.001	<.001	0.13
Model 2	0.88 (0.85-0.91)	<.001	<.001	0.26
Anxiety				
Model 0	1.25 (1.22-1.27)	<.001	<.001	0.05
Model 1	1.27 (1.24-1.30)	<.001	<.001	0.08
Model 2	1.02 (0.99-1.04)	.21	NA	0.25
Depression^c^				
Model 0	1.35 (1.33-1.38)	<.001	<.001	0.10
Model 1	1.37 (1.34-1.40)	<.001	<.001	0.13
Model 2	1.08 (1.05-1.10)	<.001	<.001	0.25
EHR helpfulness^c^				
Model 0	0.83 (0.82-0.85)	<.001	<.001	0.04
Model 1	0.84 (0.82-0.85)	<.001	<.001	0.07
Model 2	0.95 (0.93-0.97)	<.001	<.001	0.25
EHR hassles				
Model 0	1.13 (1.11-1.15)	<.001	<.001	0.03
Model 1	1.15 (1.12-1.17)	<.001	<.001	0.07
Model 2	1.04 (1.02-1.06)	<.001	.01	0.24

Abbreviations: EHR, electronic health record; NA, not applicable.

^a^ The number of survey respondents who provided responses to measures of each independent variable varied widely, as reported here. This is because each participating organization had flexibility in selecting the survey assessments they wished to include, other than the minimum required assessments of burnout, professional fulfillment, and intention to leave.

^b^ Model 0 was unadjusted. Model 1 was adjusted demographics. Model 2 was adjusted for demographics, burnout, and professional fulfillment. Model 2a was adjusted for demographics and professional fulfillment. Model 2b was adjusted for demographics and burnout.

^c^ Significant at a Bonferroni-corrected threshold of *P* < .001.

## Discussion

In a national cohort of more than 18 000 academic medical center–affiliated attending physicians representing 53 specialties, 32.6% indicated moderate or higher ITL within 2 years. ITL varied by age, gender, race, and specialty. The proportion of survey respondents meeting criteria for burnout and professional fulfillment were 37.9% and 39.3%, respectively, and varied by gender, age, race, and specialty. Burnout and lack of professional fulfillment were strongly associated with higher ITL. After adjustment for gender, age, race, specialty, burnout, and low professional fulfillment, a number of individual and institutional factors were significantly associated with ITL. Supportive leadership behaviors, personal-organizational values alignment, peer support, perceived gratitude, COVID-19 organizational support, and EHR helpfulness were associated with lower ITL, whereas depression and negative impact of work on personal relationships were associated with higher ITL.

Our finding that professional fulfillment is significantly associated with ITL expands the literature in this area. Although prior reports have also suggested that burnout is significantly associated with increased risk of ITL, there are limited data regarding other factors associated with ITL. One report indicated childcare stress was associated with increased ITL in health care workers during the COVID-19 pandemic.^19^ A recent report used machine learning to create an algorithm predicting the probability of physician departure within the next 6 months using physician characteristics, clinical productivity metrics, and data regarding electronic medical record usage in 319 nonteaching physicians working in an ambulatory practice setting.^20^ Investigators identified a number of variables that predicted physician departure, including the time since the physician was hired, the complexity of their patient panel, their age, the average proportion of appointment slots that were filled, and the time spent on clinical documentation and on the clinical inbox. Notably, the association between these factors and physician departure was nonlinear and a number of interactions were noted. For example, individuals employed for the longest and shortest durations had a higher risk of departure as compared with those with employment durations in the mid-range. The association between time spent on the EHR and physician departure varied with employment duration, with individuals with longer tenure having an increased risk of departure with longer EHR-usage duration, whereas individuals with shorter employment duration had a lower risk of departure with longer EHR-usage duration, highlighting the challenges of developing effective interventions to prevent physician departure.

Our study also provides novel information about the prevalence of ITL, burnout, and professional fulfillment across medical subspecialties in the period of 2019 to 2021, coinciding with the initial wave of the COVID-19 pandemic. This timing potentially contributes to high levels of burnout and ITL seen in specialties such as anesthesia, pulmonology, and emergency medicine. These findings are relevant to predictions of substantial shortages in emergency medicine and other front-line medical specialties, as well as the growing proportion of unfilled residency slots in some areas. Focused efforts will be needed to help support physicians working in these specialties and to attract trainees to enter these fields.

### Limitations

Our study has a number of limitations. Data collection was limited to institutions participating in the PWAC, and thus may not be representative of other academic medical centers or of physicians practicing in nonacademic settings. Although our sample size was robust with more than 18 000 respondents, our response rate was only 50.7%. Although this response rate is greater than other national physician surveys on similar topics,^21,22,23^ it is possible that our sample could have been biased to include individuals with higher levels of burnout, who may have been more inclined to complete a survey focused on this topic, or conversely, could have included individuals with higher levels of engagement with their institution. However, recent nationwide surveys of physician burnout that included secondary surveys of nonrespondents found no statistically significant difference in burnout scores between respondents and nonrespondents, suggesting that the degree of burnout may not significantly influence participation in a survey.^21,24^ Our data collection also did not include an assessment of what respondents who indicated ITL planned to do after leaving (eg, retire, leave medicine, move to a different academic or nonacademic practice setting), limiting the utility of these data in shaping interventions to help reduce physician turnover. Our data set also precluded a nuanced analysis of the prevalence or drivers of ITL by race or gender identification due to small numbers of survey respondents in some groups. Finally, our data were collected between 2019 and 2021, overlapping with the early phases of the COVID-19 pandemic. The status of COVID-19 at participating institutions could have influenced survey responses, but we are unable to adjust for these factors given that PWAC policies preclude linking survey responses to individual institutions.

## Conclusion

In this large national cross-sectional study of academic physicians, approximately one-third of survey respondents reported moderate or greater ITL. Both burnout and professional fulfillment were strongly associated with ITL. Even after adjusting for these factors, a number of personal and institutional factors were also associated with ITL. These results underscore the importance of the connections between academic physicians and both institutional leadership and mission, as well as point to the need for developing initiatives with a comprehensive approach that considers burnout, professional fulfillment, and other organizational and individual level well-being factors to help prevent physician turnover.

## Notes

Supplement 1. **eMethods.** Supplemental Methods **eTable 1.** Participating Institutions **eTable 2. **Responses by Organization **eTable 3.** Response Rate by Measure **eTable 4.** Prevalence of Intent to Leave by Specialty **eTable 5.** Prevalence of Burnout and Professional Fulfillment by Specialty **eFigure.** Consort Diagram

Supplement 2.
Data Sharing Statement
