# Supplementary material for: Well-Being Parameters and Intention to Leave Current Institution Among Academic Physicians
Source: JAMA Netw Open. 2023 Dec 15;6(12):e2347894. doi: 10.1001/jamanetworkopen.2023.47894 (PMC10724765; doi:10.1001/jamanetworkopen.2023.47894)
Supplement: Supplement 1. — eMethods. Supplemental Methods eTable 1. Participating Institutions eTable 2. Responses by Organization eTable 3. Response Rate by Measure eTable 4. Prevalence of Intent to Leave by Specialty eTable 5. Prevalence of Burnout and Professional Fulfillment by Specialty eFigure. Consort Diagram [file jamanetwopen-e2347894-s001.pdf]

Supplemental Online Content

Ligibel JA, Goularte N, Berliner JI, et al. Well-being parameters and intention to leave current institution among academic physicians. *JAMA Netw Open.* 2023;6(12):e2347894. doi:10.1001/jamanetworkopen.2023.47894

eMethods. Supplemental Methods

eTable 1. Participating Institutions

eTable 2. Responses by Organization

eTable 3. Response Rate by Measure

eTable 4. Prevalence of Intent to Leave by Specialty

eTable 5. Prevalence of Burnout and Professional Fulfillment by Specialty

eFigure. Consort Diagram

This supplemental material has been provided by the authors to give readers additional information about their work.

© 2023 Ligibel JA et al. *JAMA Network Open.*

eMethods. Supplemental Methods:

Professional Fulfillment Index (PFI) Methodology

Burnout: The PFI burnout measure assesses the two major components of physician burnout: work exhaustion and interpersonal disengagement. [1] The 10-item measure uses a 5-point Likert scale (0-4), with response options ranging from "Not at all" to "Extremely". The 0-4 scale is subsequently normalized to 0-10. Burnout is dichotomized using a published cut-point, established using two scales from the Maslach Burnout Inventory as a comparitor,[1,2]such that individuals with a score of ≥ 3.325 out of 10 are categorized as having burnout.

***Professional Fulfillment*:** The PFI professional fulfillment measure assesses happiness, meaningfulness, satisfaction, and self-efficacy, as well as a sense of worthwhileness and contributing meaningfully at work.[1] The 6-item measure uses a 5-point Likert scale (0-4), with response options ranging from "Not at all true" to "Completely True" The 0-4 scale is subsequently normalized to 0-10. Professional Fulfillment is dichotomized based on a previously established and published cut-point, such that individuals with a score of ≥ 7.5 out of 10 are categorized as being professionally fulfilled. [1]

Intent to Leave: **Intention to leave one's institution within 2 years was assessed through a 1-item Likert scale (0-4) with response options ranging from** "None" to "Definitely"[3] A score of 2 or higher (corresponding to a response of "moderate", "likely", or "definitely") was defined as a moderate or higher intent to leave.

1. Trockel M, Bohman B, Lesure E, et al. A Brief Instrument to Assess Both Burnout and Professional Fulfillment in Physicians: Reliability and Validity, Including Correlation with Self-Reported Medical Errors, in a Sample of Resident and Practicing Physicians. *Acad Psychiatry*. 2018;42(1):11-24. doi:10.1007/s40596-017-0849-3
2. Brady KJS, Ni P, Carlasare L, et al. Establishing Crosswalks Between Common Measures of Burnout in US Physicians. *J Gen Intern Med*. 2022;37(4):777-784. doi:10.1007/s11606-021-06661-4
3. Hamidi MS, Bohman B, Sandborg C, et al. Estimating institutional physician turnover attributable to self-reported burnout and associated financial burden: a case study. *BMC Health Serv Res*. 2018;18(1):851. doi:10.1186/s12913-018-3663-z

© 2023 Ligibel JA et al. *JAMA Network Open.*

eTable 1. Participating Institutions

Boston Medical Center
Children’s National Hospital
Children’s Hospital of Philadelphia
Cleveland Clinic
Dana-Farber Cancer Institute
Johns Hopkins Medicine
Mass General Brigham
Memorial Sloan Kettering Cancer Center
Michigan State University
Stanford Medicine
UMass Memorial Healthcare and the University of Massachusetts Medical School
University of New Mexico
University of Pittsburgh Medical Center
University of Wisconsin Health
Yale New Have Health

© 2023 Ligibel JA et al. *JAMA Network Open.*

eTable 2. Responses by Organization

Organization (De-identified)	No. responses (N=19,532)
1	1476
2	953
3	110
4	3262
5	2275
6	1124
7	3776
8	507
9	375
10	1861
11	773
12	256
13	669
14	1047
15	1068

© 2023 Ligibel JA et al. *JAMA Network Open.*

eTable 3. Response Rate by Measure

Outcome Measures	Original scale and response options	No. responses with ≥75% of items in the measure
**Professional Fulfillment** (6-items)	Likert scale, 0 (Not at all true) - 4 (Completely true)	18571
**Burnout** (10-items)	Likert scale, 0 (Not at all) - 4 (Extremely)	18217
**Intent to Leave** (1-item)	Likert scale, 0 (None) - 4 (Definitely)	15890
**Determinants**		
**Supportive Leadership Behaviors** (9-items)	Likert scale,<br>Items 1-8: 0 (Strongly disagree) - 4 (Strongly agree)<br>Item 9: 0 (Very dissatisfied) - 4 (Very satisfied)	17454
**Personal-Organizational Values Alignment** (3-items)	Likert scale, 0 (Not at all true) - 4 (Completely true)	17649
**Self-Valuation** (4-items)	Likert scale, 0 (Never) - 4 (Always)	14494
**Negative Impact of Work on Personal Relationships** (4-items)	Likert scale, 0 (Not at all true) - 4 (Completely true)	14156
**Depression** (4-items)	Likert scale, 0 (Never) - 4 (Always)	14360
**Anxiety** (4-items)	Likert scale, 0 (Never) - 4 (Always)	14282
**EHR Helpfulness** (4-items)	Likert scale, 0 (Never) - 4 (Always)	17217
**EHR Hassles** (3-items)	Likert scale, 0 (Never) - 4 (Always)	9904
**Control over Schedule** (5-items)	Likert scale, 0 (No control) - 4 (Complete control)	14605
**Peer Support** (4-items)	Likert scale, 0 (Not at all true) - 4 (Completely true)	10442
**Sleep-Related Impairment** (4-item)	Likert scale, 0 (Not at all) - 4 (Very much)	12509
**Meaningfulness of Clinical Work** (4-items)	Likert scale, 0 (Not at all true) - 4 (Completely true)	8554
**(COVID) Organizational Support** (9-items)	Likert scale, 0 (Not at all) - 4 (Very much)	6643
**Perceived Gratitude** (4-items)	Likert scale, 0 (Not at all) - 4 (Extremely)	9707

© 2023 Ligibel JA et al. *JAMA Network Open.*

eTable 4. Prevalence of Intent to Leave by Specialty

Specialty	N	Percent with Moderate or Higher Intent to Leave	95% CI
Allergy and Immunology	92	28.3%	(18.9% , 37.6%)
Anesthesiology	539	46.8%	(42.5% , 51.0%)
Cardiovascular Disease	369	36.3%	(31.4% , 41.2%)
Child And Adolescent Psychiatry	180	33.9%	(26.9% , 40.9%)
Colon and Rectal Surgery	41	29.3%	(14.7% , 43.8%)
Critical Care	256	39.8%	(33.8% , 45.9%)
Dermatology	225	28.9%	(22.9% , 34.9%)
Emergency Medical Services	145	26.2%	(19.0% , 33.5%)
Emergency Medicine	511	27.4%	(23.5% , 31.3%)
Endocrinology, Diabetes and Metabolism	170	27.1%	(20.3% , 33.8%)
Family Medicine	752	30.1%	(26.8% , 33.3%)
Gastroenterology	218	41.3%	(34.7% , 47.9%)
Geriatric Medicine - All Specialties	159	30.8%	(23.6% , 38.1%)
Hematology/Oncology	540	34.3%	(30.2% , 38.3%)
Hospice and Palliative Medicine - All Specialties	140	22.9%	(15.8% , 29.9%)
Hospital Medicine	100	29.0%	(20.0% , 38.0%)
Infectious Disease	203	31.5%	(25.1% , 38.0%)
Internal Medicine	1782	34.4%	(32.2% , 36.6%)
Maternal–Fetal Medicine	88	27.3%	(17.8% , 36.8%)
Medical Genetics and Genomics	49	38.8%	(24.6% , 52.9%)
Neonatal-Perinatal Medicine	245	24.9%	(19.4% , 30.4%)
Nephrology	144	37.5%	(29.5% , 45.5%)
Neurological Surgery	95	40.0%	(30.0% , 50.0%)
Neurology	467	34.9%	(30.6% , 39.2%)
Neuroradiology	124	22.6%	(15.1% , 30.0%)
Nuclear Medicine	22	13.6%	(0.0% , 29.2%)
Obstetrics and Gynecology	499	33.3%	(29.1% , 37.4%)
Ophthalmology	227	28.6%	(22.7% , 34.6%)
Orthopaedic Surgery	310	29.0%	(24.0% , 34.1%)
Otolaryngology	175	37.7%	(30.5% , 45.0%)
Pain Medicine - All Specialties	107	33.6%	(24.5% , 42.7%)
Pathology/Lab Medicine	510	30.0%	(26.0% , 34.0%)
Pediatric Anesthesiology	167	36.5%	(29.1% , 43.9%)
Pediatric Cardiology	125	26.4%	(18.6% , 34.2%)
Pediatric Critical Care Medicine	101	23.8%	(15.3% , 32.2%)

© 2023 Ligibel JA et al. *JAMA Network Open.*

Pediatric Emergency Medicine	198	26.8%	(20.5% , 33.0%)
Pediatric Hematology-Oncology	197	32.5%	(25.9% , 39.1%)
Pediatric Hospital Medicine	195	23.1%	(17.1% , 29.0%)
Pediatrics	1030	25.4%	(22.8% , 28.1%)
Pediatrics - Other Subspecialty	129	33.3%	(25.1% , 41.6%)
Physical Medicine and Rehabilitation	127	17.3%	(10.7% , 24.0%)
Plastic Surgery	80	28.8%	(18.6% , 38.9%)
Psychiatry	391	33.8%	(29.1% , 38.5%)
Psychiatry - Other Subspecialty	95	30.5%	(21.1% , 40.0%)
Pulmonary Disease	215	37.7%	(31.1% , 44.2%)
Radiation Oncology	142	31.7%	(23.9% , 39.4%)
Radiology	274	39.8%	(33.9% , 45.6%)
Radiology - Other Subspecialty	359	26.2%	(21.6% , 30.8%)
Rheumatology	102	35.3%	(25.9% , 44.7%)
Surgery	332	33.1%	(28.0% , 38.2%)
Surgery - Other Subspecialty	153	34.6%	(27.0% , 42.3%)
Thoracic Surgery	87	40.2%	(29.7% , 50.7%)
Urology	166	35.5%	(28.2% , 42.9%)

© 2023 Ligibel JA et al. *JAMA Network Open.*

eTable 5. Prevalence of Burnout and Professional Fulfillment by Specialty

		Professional Fulfillment				Burnout		
**Specialty**	N	% of N with Professional Fulfillment present	Mean (high score favorable)	SD	N	% of N with Burnout Present	Mean (low score favorable)	SD
Allergy and Immunology	100	41.0%	6.30	2.24	101	29.7%	2.76	2.01
Anesthesiology	624	34.0%	6.14	2.27	625	43.0%	3.20	2.05
Cardiovascular Disease	395	39.7%	6.44	2.19	389	36.5%	2.77	1.94
Child And Adolescent Psychiatry	211	37.0%	6.60	1.86	212	41.0%	2.99	1.86
Colon and Rectal Surgery	44	45.5%	6.84	1.95	44	31.8%	2.91	2.00
Critical Care	277	41.5%	6.48	2.27	274	39.1%	3.25	2.02
Dermatology	263	48.7%	6.93	2.07	263	32.7%	2.80	2.07
Emergency Medical Services	157	42.7%	6.84	2.18	157	40.1%	2.95	2.08
Emergency Medicine	565	35.6%	6.31	2.09	561	42.1%	3.20	1.94
Endocrinology, Diabetes and Metabolism	180	33.9%	6.52	1.91	182	33.0%	2.79	1.91
Family Medicine	788	34.8%	6.26	2.15	783	42.3%	3.18	2.03
Gastroenterology	242	36.0%	6.22	2.13	237	38.8%	3.06	2.05
Geriatric Medicine - All Specialties	169	43.8%	6.70	2.05	167	35.3%	2.76	1.91
Hematology/Oncology	574	43.9%	6.83	2.02	576	41.7%	3.14	1.90
Hospice and Palliative Medicine - All Specialties	150	49.3%	7.15	2.00	149	36.2%	2.95	1.88
Hospital Medicine	114	39.5%	6.48	2.01	114	41.2%	3.04	2.09
Infectious Disease	221	44.3%	6.67	2.09	218	37.2%	2.92	1.73
Internal Medicine	1893	35.8%	6.31	2.21	1890	40.2%	3.06	2.05
Maternal – Fetal Medicine	97	43.3%	6.84	1.85	98	39.8%	2.90	1.65
Medical Genetics and Genomics	58	41.4%	6.51	1.94	57	42.1%	3.07	1.68
Missing	2827	37.1%	6.32	2.17	2566	40.0%	3.01	1.99
Neonatal - Perinatal Medicine	282	46.1%	6.91	1.84	279	26.5%	2.49	1.73
Nephrology	157	43.3%	6.65	2.09	158	41.8%	2.99	1.93
Neurological Surgery	115	47.8%	6.95	2.36	116	29.3%	2.46	2.14
Neurology	535	37.9%	6.52	2.05	531	39.2%	2.96	1.95
Neuroradiology	129	46.5%	6.69	1.87	128	26.6%	2.30	1.76
Nuclear Medicine	26	42.3%	7.28	1.33	25	24.0%	2.14	1.73
Obstetrics and Gynecology	529	36.7%	6.43	2.03	532	43.2%	3.22	1.95
Ophthalmology	256	46.5%	6.94	1.98	255	32.9%	2.62	1.88
Orthopaedic Surgery	358	48.0%	6.91	2.11	353	31.4%	2.62	1.92

© 2023 Ligibel JA et al. *JAMA Network Open.*

Otolaryngology	208	38.5%	6.45	2.21	210	45.2%	3.20	2.13
Pain Medicine - All Specialties	121	37.2%	6.38	2.31	122	27.9%	2.53	1.78
Pathology/Lab Medicine	574	44.1%	6.77	2.13	544	28.9%	2.45	1.87
Pediatric Anesthesiology	204	39.2%	6.49	2.16	206	38.3%	3.21	2.08
Pediatric Cardiology	177	40.7%	6.71	1.99	177	31.6%	2.68	1.74
Pediatric Critical Care Medicine	125	40.0%	6.59	2.13	125	35.2%	2.89	1.78
Pediatric Emergency Medicine	207	34.8%	6.44	1.80	208	31.7%	2.80	1.73
Pediatric Hematology - Oncology	224	42.0%	6.76	1.85	222	36.9%	2.74	1.75
Pediatric Hospital Medicine	242	38.8%	6.55	1.92	238	30.3%	2.68	1.77
Pediatrics	1189	37.8%	6.55	1.95	1183	36.6%	2.90	1.81
Pediatrics - Other Subspecialty	145	35.9%	6.54	1.88	145	28.3%	2.83	1.93
Physical Medicine and Rehabilitation	131	41.2%	6.78	1.95	130	29.2%	2.61	1.79
Plastic Surgery	100	48.0%	6.96	1.94	99	34.3%	2.86	2.07
Psychiatry	430	40.2%	6.53	2.05	433	42.3%	3.14	1.94
Psychiatry - Other Subspecialty	110	42.7%	6.80	2.06	111	32.4%	2.65	1.83
Pulmonary Disease	230	34.3%	6.22	2.17	221	45.2%	3.17	1.95
Radiation Oncology	160	54.4%	7.10	2.14	160	36.9%	3.01	2.08
Radiology	315	33.7%	6.15	2.26	314	38.9%	3.03	1.86
Radiology - Other Subspecialty	396	39.6%	6.52	2.15	391	30.7%	2.65	1.89
Rheumatology	107	32.7%	6.34	2.25	106	39.6%	2.85	1.90
Surgery	375	44.0%	6.88	2.12	374	36.9%	2.87	1.99
Surgery - Other Subspecialty	170	51.8%	6.92	1.98	168	35.1%	2.73	2.09
Thoracic Surgery	109	44.0%	6.76	2.25	109	38.5%	2.75	2.09
Urology	186	45.7%	6.73	2.36	181	42.0%	3.03	2.15

© 2023 Ligibel JA et al. *JAMA Network Open.*

eFigure. Consort Diagram

Response Criteria is met by answering ≥75% of items for one or more outcome measure (Professional Fulfillment, Burnout, or Intent to Leave)

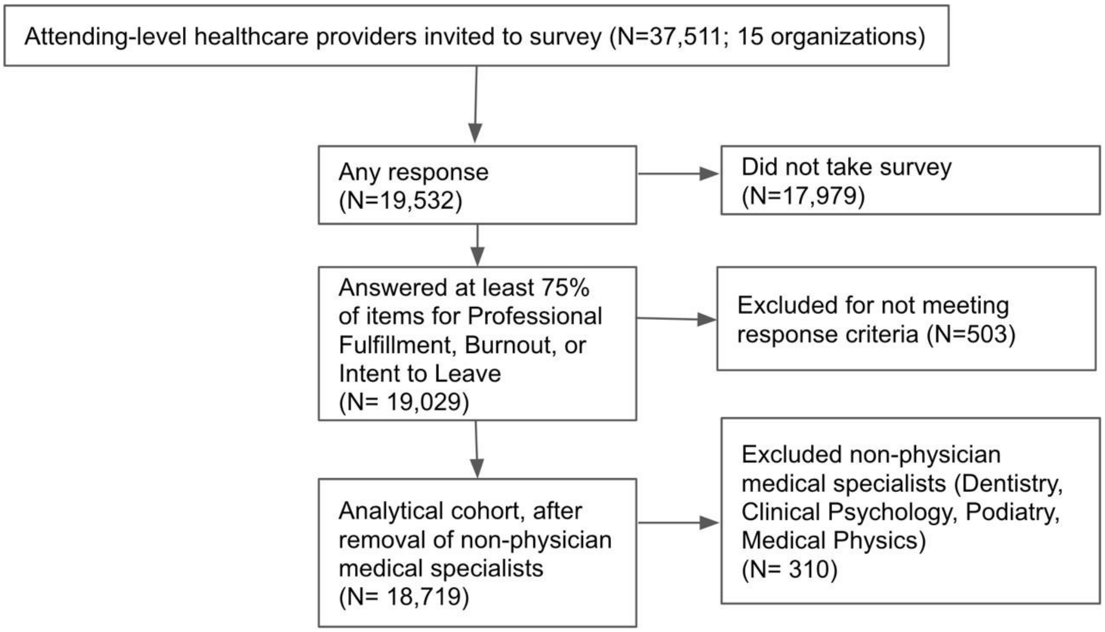

© 2023 Ligibel JA et al. *JAMA Network Open.*
