# Supplementary material for: Well-Being Parameters and Intention to Leave Current Institution Among Academic Physicians
Source: JAMA Netw Open. 2023 Dec 15;6(12):e2347894. doi: 10.1001/jamanetworkopen.2023.47894 (PMC10724765; doi:10.1001/jamanetworkopen.2023.47894)
Supplement: Supplement 2. — Data Sharing Statement [file jamanetwopen-e2347894-s002.pdf]

Data Sharing Statement

Ligibel. Well-Being Parameters and Intention to Leave Current Institution Among Academic Physicians. *JAMA Netw Open*. Published December 15, 2023.
doi:10.1001/jamanetworkopen.2023.47894

Data
Data available: No

Additional Information
Explanation for why data not available: Data sharing agreements with the organizations who participate in the Healthcare Professional Well-being Academic Consortium do not allow us make the data used for this study publicly available.
